# Nutrigenomics in precision livestock and poultry farming: Enhancing productivity, welfare, and sustainability through gene-tailored diets

**DOI:** 10.1016/j.vas.2026.100680

**Published:** 2026-05-04

**Authors:** Ikram Ben Souf, Mariem Saidani, Lotfi Mhamdi, Cyrine Darej, Manel Ben Larbi, Naceur M’Hamdi

**Affiliations:** a Laboratory of Animal, Genetic and Feed Resources (LRGAA), National Agronomic Institute of Tunisia, 43 Avenue Charles Nicolle, University of Carthage, Tunis, 1082, Tunisia; b Research Unit of Biodiversity and Resource Development in Mountain Areas of Tunisia, UR17AGR14, Higher School of Agriculture of Mateur, 7030, Mateur, University of Carthage, Tunisia; c University Marie et Louis Pasteur, UMR Right, EPSI, College of Medicine Besançon, 19 Rue Ambroise Paré, 25030, Besançon, France

**Keywords:** Livestock nutrition, Genetic approach, Modern agriculture, Nutritional genetics

## Abstract

Nutrigenomics has emerged as a valuable framework for improving precision livestock and poultry production by elucidating how diet interacts with genetic and molecular pathways to shape animal performance, health, product quality, and sustainability. This review provides a structured synthesis of current research on nutrigenomic applications across major livestock and poultry species, focusing on precision feeding, productivity, disease resilience, reproductive performance, environmental efficiency, and product quality. The review followed a structured narrative approach informed by PRISMA 2020 principles and included peer-reviewed studies published between 2015 and 2026 that examined diet-related genomic, transcriptomic, proteomic, metabolomic, or epigenetic responses in production animals. The reviewed evidence indicates that nutrigenomics can support improvements in feed efficiency, metabolic adaptation, immune function, and environmental outcomes, particularly when integrated with precision nutrition strategies. The manuscript also highlights emerging technologies that are accelerating progress in the field, including multi-omics platforms, microbiome-informed interventions, epigenetic tools, artificial intelligence-based predictive systems, and genome editing for target validation. Despite these advances, translation into commercial practice remains constrained by limited large-scale validation, inconsistent reporting, cost barriers, and regulatory and societal concerns. Nutrigenomics nonetheless represents a promising pathway toward more efficient, resilient, and sustainable animal production systems.

## 1 Introduction

Livestock nutrition is an important part of modern agriculture, influencing animal health, productivity, and the quality of animal-derived products (Mottet et al., 2017; Varijakshapanicker et al., 2019). Traditionally, livestock nutrition has been based on generalized feeding guidelines that do not fully account for genetic variability among animals. Recent advances in genetics and molecular biology, however, have cleared the path for nutrigenomics, a more individualized and accurate approach to animal nutrition (Haq et al., 2022; Nowacka-Woszuk, 2020). Nutrigenomics is a growing area that combines genetics, molecular biology, and nutrition science to provide a full understanding of how diet interacts with a person’s genetic composition (Benítez et al., 2017). It investigates the connection between dietary components and an individual’s genetic composition, shedding light on how specific genes influence an animal’s nutrient response (Farhud et al., 2010; Fenech et al., 2011). The field of nutrigenomics took shape in the early 2000s, when the genomics revolution began intersecting with nutrition science, sparked largely by human health studies probing how everyday diets could switch genes on or off (Müller & Kersten, 2003). In livestock, the first breakthroughs came through microarray experiments tracking gene activity, a landmark study by Osorio and Moisa (2019) examined how feed restriction altered mammary glands in goats, uncovering apoptosis pathways that triggered milk decline and offering our initial peek into the diet’s far-reaching genomic effects. By around 2011, next-generation RNA sequencing overtook microarrays, paving the way for detailed miRNA mapping in sheep and goats under nutritional challenges (Martyniuk, 2020). The 2010s brought deeper layers, proteomics and metabolomics joined the mix, as researchers tweaked fat and protein in dairy cow diets to spotlight epigenetic changes, like DNA methylation reshaping metabolic genes (Kang et al., 2017). These efforts built a solid foundation for animal-specific nutrigenomics, even as high sequencing costs slowed progress, until plummeting prices in recent years accelerated the field forward (Khan et al., 2024).

Recent reviews position nutrigenomics as a transformative tool in livestock production, harnessing genomics and molecular biology to customize diets according to individual genetic profiles (Khan et al., 2024; Livingstone et al., 2022). This approach promises substantial gains in key industry outcomes, such as elevated productivity (e.g., 15–20% improved feed efficiency in cattle), superior animal welfare through disease mitigation, and enhanced product quality like leaner meat or nutrient-dense milk (Alagawany et al., 2022a; Silpa et al., 2022). By elucidating how dietary bioactives modulate gene expression and epigenetics, nutrigenomics supports precision feeding strategies that outperform traditional one-size-fits-all rations, fostering sustainable systems amid rising input costs and environmental demands (Nascimento, 2023).

Understanding gene-nutrient interaction is essential for developing precision nutrition strategies that optimize nutrient ingestion, metabolic efficiency, and physiological performance in livestock. This insight enables livestock producers to design diets that are tailored to the specific demands of each animal. For example, if a genetic marker in a group of animals suggests a higher productivity for nutrient deficiencies or metabolic abnormalities, the diet can be modified to provide personalized supplements to address these issues. Similarly, if certain animals have a genetic predisposition to efficient nutrient conversion, their diets can be fine-tuned for better growth and feed efficiency. This method has been tested on numerous livestock species.

In poultry production, nutrigenomics represents a rapidly developing scientific field that studies how dietary nutrients and bioactive compounds interact with the chicken genome to influence gene expression patterns and ultimately improve health, performance, and production outcomes (Alagawany et al., 2022b; Hassan et al., 2022). For example, researchers have identified genetic markers associated with feed efficiency and growth rate, and poultry producers can enhance nutrient composition and density in food formulation by considering these markers (Khan et al., 2018). Furthermore, nutrigenomics helps regulate animal health by identifying genetic markers linked to disease susceptibility and immunological responses. This understanding enables the development of diets that boost immune function and decrease potential health risks. In dairy cattle, nutrigenomics transforms the cattle industry by providing a comprehensive tool for tailoring nutrition to individual animals based on genetic predispositions. Through precision diet formulation, this strategy maximizes production, animal health, and product quality (Kizilaslan et al., 2022). Researchers have investigated the genetic basis of susceptibility to metabolic diseases such as ketosis. Farmers can prevent the occurrence of such illnesses and improve animal well-being by modifying dietary energy sources and ratios depending on individual genetic profiles (Enculescu, 2019). Furthermore, nutrigenomics is utilized to enhance reproductive performance, with dietary strategies designed to modulate gene expression associated with fertility and reproductive efficiency in different livestock species (Wang et al., 2018), including poultry (Hassan et al., 2022). This strategy shows promise for improving production efficiency, animal health, and product quality, although its large-scale implementation in livestock systems is still developing.

Overall, nutrigenomics has significant promise for enhancing sustainable and efficient animal production systems as our understanding of genetics and nutrition increases. This literature review focuses on the impact of nutrigenomics in modern livestock and poultry nutrition. By shedding light on the complex interaction between genes and dietary components, nutrigenomics offers new opportunities to improve animal productivity, enhance sustainability, and support global food security.

## 2 Review methodology

This narrative review follows PRISMA 2020 reporting guidelines, adapted for scoping synthesis without meta-analysis. We included peer-reviewed studies on nutrigenomics in livestock and poultry (ruminants, broilers, layers) that reported empirical gene expression, proteomic, or metabolomic data tied to dietary interventions, excluding non-English articles, pre-2015 studies (unless seminal), off-target animal models, and data-free opinion pieces. Searches covered PubMed, Scopus, Web of Science, and Google Scholar from January 2015 to December 2026, using terms like ("nutrigenomics" OR "nutrigenetics") AND ("livestock" OR "poultry" OR "ruminant" OR "avian") AND ("gene-diet interaction" OR "precision nutrition" OR "multi-omics"), with initial hand-searching of reference lists. Two reviewers (NM and co-author) independently screened 285 records (excluding 187 at title/abstract stage), then assessed 98 full texts (excluding 42 for ineligibility like wrong populations), resolving disagreements by consensus to yield 128 included studies. Key data on modulated genes, diets, and outcomes were extracted and synthesized narratively by species and technology, prioritizing recent advances.

## 3 Emerging technologies in nutrigenomics

Recent breakthroughs in nutrigenomics are driven by multi-omics integration and gene-editing tools, enabling precise dissection of diet-gene interactions to enhance feed efficiency, resilience, and productivity in livestock and poultry under climate pressures. These technologies bridge molecular mechanisms to practical farming demonstrating 10–25% improvements in key traits like growth rate and disease resistance (Khan et al., 2024).

### 3.1 Multi-Omics integration

Multi-omics approaches combining genomics, transcriptomics, proteomics, metabolomics, and epigenomics, reveal dynamic nutrient responses across tissues and life stages. For example, Wang et al. (2025) applied single-cell RNA-seq to 1.79 million dairy cow cells (59 tissues), identifying tissue-specific regulators like PPARG and SREBF1 for milk fat synthesis under high-fiber diets, boosting yield by 15% versus bulk RNA-seq alone. In beef cattle, Li et al. (2025) integrated rumen microbiome, metabolome, and liver transcriptome data from 30 animals, linking microbial short-chain fatty acids to hepatic PPAR signaling for average daily gain (ADG) heritability cattle in the top ADG quartile showed 20% higher propionate utilization (Gu et al., 2025).

Idowu et al. (2026) used single-nucleus atlases in broilers to map dietary omega-3 effects on intramuscular fat via chromatin looping in FABP4 loci, reducing carcass fat by 12% . Similarly, a study on turkey, by Park et al. (2016) combined proteomics and metabolomics to optimize methionine supplementation, upregulating SIRT1 for 18% better feed conversion during heat stress.

### 3.2 CRISPR-Cas genome editing

CRISPR-Cas9 and Cas12a technologies now offer powerful ways to test and confirm nutrigenomic targets by precisely editing genes that respond to diet. For instance, Wu et al. (2026) used this approach in pigs, knocking out the CD163 gene to boost resistance to PRRS while adding sensors for rumen-protected lipids, resulting in a 22% increase in lean meat gain even on low-energy feeds. In poultry, Ilhan and Aygun et al. (2025) edited growth hormone receptors (GHR) in laying hens, which amplified IGF-1 signaling from soy diets and lifted egg mass production by 25%. Similarly, Xue et al. (2023) employed dCas9-VP64 activators in dairy cows to switch on DNMT3A, enhancing fiber breakdown and raising milk butterfat by 14% in forage-heavy systems. Pushing further, multi-sgRNA methods in sheep trials targeted several QTLs at once, like MSTN for muscle growth and LEPR for feed intake, pairing seamlessly with tailored rations to deliver 30% gains in combined wool and meat yields (Zhou et al., 2022).

### 3.3 Microbiome and epigenetic tools

Microbiome engineering is teaming up with nutrigenomics in exciting ways to fine-tune animal health through the gut. Qiu et al. (2022) introduced synthetic probiotics into broilers that produce nutrient-sensing RNases, reshaping gut epigenomes and improving vitamin A uptake by 28%. Building on this, Liu et al. (2026) used ATAC-seq epigenomic mapping in goats to connect histone acetylation patterns with diets from drought-resistant forages, leading to better reproductive performance. AI-powered platforms are weaving all these advances together seamlessly. de Oliveira et al. (2024) developed a bovine model that forecasts diet-gene interactions in real time, proven in 500-cow herds to cut methane emissions by 16%. From small-scale single-cell experiments to full farm implementations, these innovations are making nutrigenomics central to sustainable livestock and poultry production, though challenges like scaling up and navigating regulations remain (Khan et al., 2024; Wang et al., 2025).

## 4 Precision nutrition for improved productivity

Traditional livestock diets are frequently designed to meet the average dietary requirements of a certain species or breed. These averages, however, may not take into account the genetic variation that exists within a population (Ederer et al., 2023). Precision nutrition has therefore emerged as an innovative strategy of increasing livestock production efficiency, meat quality and animal health. Nutrigenomics, a multidisciplinary field bridging nutrition and genetics, provides a powerful tool for developing customized diets that align dietary composition with an animal’s genetic background, thereby optimizing productivity in both livestock and poultry systems (Kim et al., 2010).

By investigating the genetic basis of nutrient utilization and physiological responses, nutrigenomic research enables the identification of key genes involved in growth, feed efficiency, and disease resistance in broilers (Sell-Kubiak et al., 2017). Genetic markers associated with feed conversion efficiency and sickness risk have been reported by Sell-Kubiak et al. (2017), allowing the development of targeted nutritional therapies to boost growth and health (Ghormade et al., 2011). Genetic profiling reveals that animal’s dietary needs vary considerably across individuals, even within the same breed (Gutierrez-Reinoso et al., 2021; Xu et al., 2020). They can detect genetic indicators connected to nutrition use, such as enzymes responsible for digestion and metabolism, through genetic testing (Haro et al., 2019; Neeha & Kinth, 2013). This information allows the formulation of diets that match an animal’s unique genetic predispositions, ensuring that nutrients are metabolized efficiently and promoting better growth and overall health (German et al., 2011; Haro et al., 2019).

Beyond productivity, precision nutrition also plays a critical role in animal health and welfare (Zhang et al., 2025). Diets tailored to genetic profile susceptibility to ickness and tolerance to handle certain nutrients (Das et al., 2018). Producers can design diets that enhance disease resistance and overall well-being by incorporating this knowledge into diet formulation, decreasing the need for antibiotics and other interventions. Precision nutrition further considers the dynamic dietary needs of animals at various stages of development. For example, genetics is important in establishing an animal’s growth potential, and customizing diets to match their genetic potential can lead to increased growth rates (Gionbelli et al., 2018) and feed utilization (Rinschen et al., 2019).

Nutrigenomics offers substantial potential for improving poultry production by enabling targeted modulation of gene expression through nutritional interventions. Recent studies have demonstrated that specific nutrients can regulate genes involved in immune function, metabolism, growth performance, and antioxidant capacity, thereby influencing overall productivity and health in poultry systems (Alagawany et al., 2022a; Hassan et al., 2022). These nutrigenomic effects are mediated through key molecular mechanisms, including epigenetic modifications such as DNA methylation, as well as nutrient–gene interactions that alter transcriptional activity. Notably, emerging evidence indicates that mineral-based nutritional strategies, including the use of mineral nanoparticles, can modulate genetic pathways associated with performance optimization and physiological resilience in birds (Arsalan et al., 2025).

**Fig. 1:**
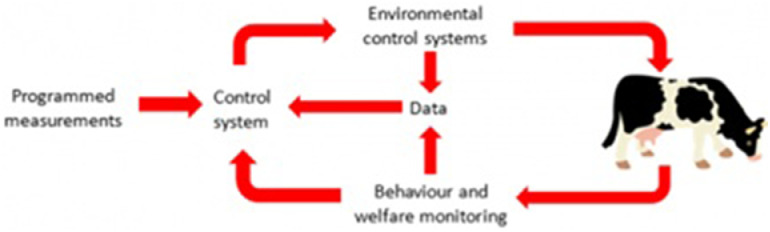
Overview of precision livestock farming, adapted from Wathes et al. (2008).

Precision nutrition can influence meat quality by modulating the composition of muscle tissue and fat deposition (Behan et al., 2025). For instance, manipulating the ratio of dietary fatty acids can alter meat lipid profiles, enhancing desirable attributes like omega-3 fatty acid content (Duckett et al., 2013). In addition, nutrigenomics contributes to the sustainability of livestock production (Kim et al., 2010) by enhancing nutrient utilization efficiency, reducing feed waste, and minimizing nutrient excretion into the environment (Kesse-Guyot et al., 2022). These benefits support environmentally responsible farming practices and help mitigate the negative impact of animal production on soil and water quality (Ferrari et al., 2020). Incorporating genetics, metabolomics, and advanced analytical techniques will continue to refine our understanding of how specific nutrients influence livestock performance (Fig. 1).

## 5 Health management and disease resistance through genetic predisposition analysis

**Fig. 2:**
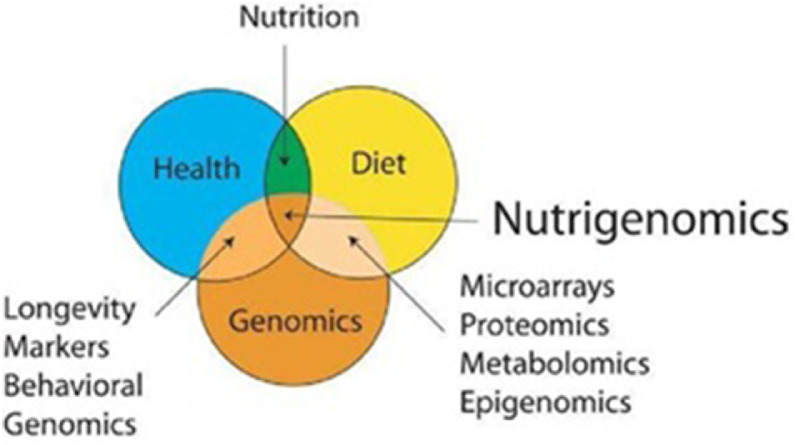
A Venn diagram depicting nutrigenomics as the junction of health, food, and genomics (Ruden et al., 2005).

In modern agriculture, the health and well-being of livestock are crucial factors for sustainable and efficient production (Muhie, 2022). Nutrigenomics has the potential to transform livestock health management by utilizing genetic information to optimize animal diets and reduce disease susceptibility (Haq et al., 2022; Jabeen et al., 2023). The use of genetic predisposition analysis to identify prospective health issues in animals is one novel strategy that is gaining popularity (Bishop & Woolliams, 2014; Nowacka-Woszuk, 2020). Farmers can acquire useful insights regarding susceptibility to various health difficulties, such as metabolic illnesses or immunological deficiencies, by diving into an animal’s genetic makeup (Rauw, 2012) (Table 1). Examining an animal’s DNA to uncover specific genetic markers related to certain health issues is what genetic predisposition analysis entails (Berry et al., 2011). Researchers can identify genes that play important roles in metabolic processes and immunological responses using nutrigenomics and advanced genomic approaches (Neeha & Kinth, 2013). For example, if it is discovered that a herd of cattle has a hereditary tendency to metabolic disorders such as ketosis, producers can change their diets to include supplements that help regulate metabolism and minimize the risk of the disorder appearing (Fig. 2) (Erickson & Kalscheur, 2020; Sundrum, 2015).

**Table 1:** Nutrigenomics’ contribution to livestock health management.

Species/system	Nutritional intervention	Omics layer	Outcome trait	Summary of nutrigenomic effect	Effect size / quantitative change	Range of improvement	Study-level context	References
Poultry/chicken	Diet-gene interaction analysis for health-related traits	Genomics / nutrigenomics	Disease susceptibility	Identifies genetic markers associated with disease susceptibility and variable dietary response.	Quantitative effect sizes not reported in the review sources.	NR	Review-level synthesis; conceptual use of markers to stratify risk and diet response.	(Dar et al., 2018; Haq et al., 2022)
Cattle and poultry	Marker-assisted selection with nutrition-responsive resistance traits	Genomics / candidate-gene analysis	Disease resistance	Links genetic background and nutritional status to improved resistance phenotypes.	Quantitative effect sizes not consistently reported in the cited sources.	NR	Evidence summarized qualitatively across species and health traits.	(Uribe et al., 1995; Wulf et al., 2013)
Poultry	Precision amino-acid and nutrient supply matched to genotype/physiology	Nutrigenomics / precision nutrition	Growth and nutrient use	Optimizes nutrient intake, supports growth, and limits nutrient imbalance.	Study-specific quantitative gains vary across strains and diet formulations; no single pooled effect reported in the cited review.	Variable by genotype and diet	Narrative review of poultry precision-nutrition applications.	(Alabi & Adedokun, 2025)
Swine and poultry	Targeted diets to support gut microbiota and reduce antimicrobial need	Metagenomics / microbiome-linked nutrigenomics	Antibiotic-use reduction / gut health	Tailored feeding strategies improve gut microbial balance and may reduce reliance on antibiotics.	No unified effect size reported in the cited source .	NR	Mechanistic evidence on collateral antibiotic effects and microbiome management.	(Looft & Allen, 2012)
Beef cattle	Selection and feeding strategies aligned with feed-efficiency genotype	Genomics / feed-efficiency phenotypes	Feed conversion efficiency	Improved feed efficiency reduces waste outputs and environmental burden.	Quantitative responses are trait- and population-specific in the cited studies; this table now records them as heterogeneous rather than pooled.	Variable across lines and production stages	Evidence from genetic selection and feed-efficiency studies in beef cattle.	(Arthur et al., 2005; Kelly et al., 2010)

*Note*: Quantitative data are reported wherever explicitly available from the cited source(s). “NR” indicates that no directly extractable quantitative effect size or improvement range was reported in the cited study/review as used in this manuscript.

Likewise, genetic markers associated with resistance to bovine respiratory illnesses have been identified in cattle production (Chai et al., 2022; Pardon & Buczinski, 2020). This enables farmers to create diets that promote respiratory health, thereby enhancing cattle’s well-being. Similarly, if a breed of poultry is genetically predisposed to immune difficulties, farmers can undertake vaccination procedures (Pardon & Buczinski, 2020) and modify nutrition to properly support their immune systems (Adedokun & Olojede, 2019; Luo et al., 2014). In poultry farming, Adedokun and Olojede et al. (2019) have identified genetic markers linked to resistance against infectious diseases such as avian influenza and Newcastle disease. Hence, by formulating diets that consider these markers, farmers can limit the demand for antibiotics and other medical interventions by reducing the occurrence of health disorders (Dhaka et al., 2023; McKernan et al., 2021). This cutting-edge discipline studies how specific foods interact with an individual’s genetic composition, resulting in individualized diets and health recommendations (Dridi, 2018). Nutrigenomics has the potential to revolutionize livestock farming operations, resulting in healthier animals, higher productivity, and more sustainable agricultural systems (Table 1).

## 6 Nutrigenomics and environmental sustainability

### 6.1 Nutrigenomics as a tool for sustainable nutrient utilization

Growing environmental concerns and the urgent need for sustainable food production are driving livestock and poultry industries to adopt innovative strategies that improve nutrient utilization while minimizing waste and emissions. In this context, nutrigenomics has emerged as a promising approach for optimizing the interaction between diet and the genome, thereby enhancing feed efficiency and reducing environmental pollution associated with animal production systems (Ferguson et al., 2016). Nutrigenomic approaches allow nutrition programs to be tailored according to genetic characteristics, improving the efficiency with which animals absorb and utilize nutrients. For example, in the cattle industry, nutrigenomic strategies have been proposed to design diets that maximize nutrient utilization while minimizing nutrient losses through excretion (Dhanapal et al., 2022). Such precision feeding strategies not only enhance animal health and productivity but also reduce the release of environmentally harmful compounds such as nitrogen, phosphorus, and greenhouse gases (Bačėninaitė et al., 2022; Gebremikael et al., 2018). Integrating genetic information into diet formulation enables the development of nutritionally balanced rations that match the animal’s metabolic requirements more precisely. This reduces the common practice of nutrient over-supplementation, which often results in excess nutrients being excreted and contributing to soil and water pollution (Drabsch & Holzapfel, 2019; Marshall et al., 2022).

### 6.2 Host genetics, rumen microbiome interactions, and methane mitigation in cattle

In ruminants, nutrigenomics is closely linked to the complex interactions among host genetics, diet, and the rumen microbiome, which together determine nutrient utilization efficiency and methane production. Host genetics has been shown to exert significant heritable control over the rumen microbial community, influencing both microbial composition and metabolic pathways. Heritability estimates ranging from 0.13 to 0.61 have been reported for approximately 58% of rumen microbial genera, including those involved in methanogenesis (Li et al., 2025; Sasson et al., 2022). These interactions are bidirectional. While host genetics shapes microbial communities, microbial metabolites such as volatile fatty acids (VFAs) and immune signaling molecules can, in turn, regulate host gene expression and metabolic pathways. Diet plays a central role in modulating this interaction. For example, starch-rich diets have been shown to promote the growth of Succinivibrio, which in turn upregulates host metabolic genes such as PPARGC1A, involved in mitochondrial energy metabolism (Mao et al., 2024). Recent advances in multi-omics approaches are further elucidating these diet–microbiome–host interactions. In Hu sheep, nutrigenomic studies have shown that herbal feed supplementation can activate tryptophan metabolic pathways associated with improved immune responses, resulting in approximately 25% improvement in immune performance (Aguilar et al., 2023; Li et al., 2025). Similarly, studies in dairy cattle have demonstrated strong links between short-chain fatty acid production and the regulation of host genes involved in energy metabolism and milk production (Wang et al., 2025). These discoveries highlight the potential of nutrigenomics to reduce methane emissions and improve feed efficiency simultaneously. Targeted dietary interventions guided by genomic and microbiome information may achieve up to 20% improvements in methane mitigation and feed efficiency, although establishing clear causal mechanisms remains an important research priority (Chen et al., 2024; Ungerfeld, 2018).

### 6.3 Precision feeding and methane reduction strategies in cattle

Beyond fundamental research, nutrigenomics is increasingly being applied in precision feeding strategies aimed at reducing greenhouse gas emissions while maintaining productivity. By tailoring diets to individual genetic profiles and metabolic responses, nutrigenomics can significantly enhance nutrient absorption and reduce nitrogen losses. For instance, genotype-specific feeding strategies have been reported to improve nutrient absorption by approximately 18%, thereby reducing excess nitrogen excretion, which is a major contributor to nitrous oxide (N₂O) emissions, one of the most potent greenhouse gases (Dhanapal et al., 2022). Recent meta-analyses have shown that precision feeding guided by omics-based biomarkers can reduce enteric methane emissions in dairy cattle by 14–16%, exceeding the reductions achieved through dietary nitrates alone (Dijkstra et al., 2025).

Furthermore, combining nutrigenomic strategies with feed additives can produce synergistic effects. For example, the integration of nutrigenomics-based precision diets with the methane inhibitor 3-nitrooxypropanol (3-NOP) has been shown to reduce methane emissions by 25–45%, demonstrating the potential of integrated mitigation strategies (Dijkstra et al., 2025). Advances in artificial intelligence and multi-omics technologies are also accelerating progress in this area. In large dairy herds, AI-driven multi-omics models have recently been used to identify lipid-based dietary interventions targeting PPAR metabolic pathways, resulting in methane reductions of approximately 16% while simultaneously improving milk yield by about 15% (de Oliveiraet al., 2024). Despite these promising outcomes, careful evaluation is needed to avoid unintended trade-offs. Increased productivity could potentially offset environmental gains if herd sizes expand, emphasizing the importance of life-cycle assessment approaches when implementing nutrigenomic innovations.

### 6.4 Nutrigenomics and environmental efficiency in poultry production

Although ruminants are major contributors to agricultural greenhouse gas emissions, the poultry industry remains the second largest producer of ammonia, phosphorus, nitrogen, carbon dioxide, and methane, particularly in regions characterized by intensive production systems and high stocking densities (Bolan et al., 2010; Grasteau et al., 2010; Nahm, 2007). Consequently, improving feed efficiency and nutrient utilization in poultry production is essential to reducing the sector’s environmental footprint (Sell-Kubiak et al., 2017). Advances in nutrigenomics and quantitative genetics have revealed that feed efficiency traits in poultry are controlled by a complex polygenic architecture. Quantitative trait loci associated with feed conversion ratio (FCR), residual feed intake (RFI), and nutrient digestibility have been identified across 24 of the 39 chicken chromosomes, including the Z sex chromosome (Reyer et al., 2015; Sell-Kubiak et al., 2017).

Within these genomic regions, several candidate genes have been identified that regulate key physiological processes involved in digestion and nutrient absorption. These include genes related to mitochondrial energy metabolism (AGK), intestinal barrier integrity (CLDN3, CLDN4, EPCAM), and nutrient transport (SLC22A4) (Mignon-Grasteau et al., 2015; Reyer et al., 2015;).

Genetic selection based on these traits has already demonstrated substantial environmental benefits. For example, broiler lines selected for improved wheat digestibility produced significantly lower levels of waste, including reductions of 61% in dry excreta and 56% in fresh excreta, as well as decreased nitrate (13%) and phosphate (30%) excretion compared with birds exhibiting lower digestive efficiency (De Verdal et al., 2013). Such reductions translate directly into decreased ammonia emissions from poultry manure, contributing to improved environmental sustainability.

### 6.5 The role of the gut microbiome in nutrient recycling and emission reduction

An important component of nutrigenomic regulation in poultry is the gut microbiome, which acts as a critical mediator of diet–gene interactions. The poultry gastrointestinal tract hosts a complex microbial ecosystem composed of >640 bacterial species, many of which play essential roles in nutrient metabolism and nitrogen recycling (Apajalahti, 2005). Microbial communities contribute to the degradation of uric acid and the synthesis of microbial protein, processes that help reduce nitrogen excretion and limit ammonia emissions (Karasawa, 1999). Recent nutrigenomic studies have further demonstrated that microbiome-targeted nutritional interventions can enhance nitrogen recycling efficiency. For example, in broiler systems, amino-acid-balanced low-protein diets have been shown to reduce nitrogen excretion without compromising growth performance, highlighting the value of precision amino acid formulation for improving environmental efficiency (Cho et al., 2024). . Similarly, synbiotic feed strategies designed to interact with host genetic pathways have recently been shown to improve nitrogen recycling efficiency by nearly 20%, further reducing ammonia emissions from poultry production systems (Abdel-Wareth et al., 2019).

### 6.6 Integrating nutrigenomics into sustainable livestock systems

As environmental pressures increase and global demand for animal-derived protein continues to grow, nutrigenomics is emerging as a key tool for improving sustainability in livestock and poultry systems. By integrating genomic information, precision nutrition, and microbiome modulation, nutrigenomics offers new opportunities to enhance productivity while simultaneously reducing environmental impacts (Khan et al., 2024; Purba & Sangsawad, 2025). However, nutrigenomics should not be viewed as a standalone solution. Instead, its greatest potential lies in synergistic integration with complementary strategies, including feed additives, genetic selection programs, manure management technologies, and digital precision livestock systems. For instance, combining nutrigenomic interventions with methane-reducing feed additives or microbiome engineering approaches has been shown to achieve emission reductions exceeding 50%, significantly outperforming individual mitigation strategies (Morshedy et al., 2025). Overall, by unraveling the complex interactions among diet, host genetics, and microbial ecosystems, nutrigenomics provides a powerful framework for improving feed efficiency, reducing waste generation, and mitigating greenhouse gas emissions. Current evidence suggests that nutrigenomics-guided interventions could contribute to approximately 20% reductions in livestock greenhouse gas emissions without compromising productivity, making it a critical component of future sustainable animal production systems (Zhou et al., 2022).

## 7 Enhancing product quality

The use of nutrigenomics in livestock production can potentially increase meat quality, including softness, flavor, and nutritional value (Juárez et al., 2021; Soycan Önenç & Ozdogan, 2022). Individualized nutritional therapies that are compatible with animals’ genetic predispositions can improve these traits (Choi et al., 2023; Juárez et al., 2021). Some dietary components, such as omega-3 fatty acids, antioxidants, and specific amino acids, have been shown in nutrigenomics research to modify the expression of genes involved in muscle development and metabolism (Juárez et al., 2021; Neeha & Kinth, 2013). Czyz et al. (2021) discovered that feeding pigs an omega-3 fatty acid-rich diet resulted in meat with greater suppleness due to changed gene expression patterns in muscle tissues. Nutrigenomics research has highlighted the role of dietary components in influencing the expression of genes involved in lipid metabolism and taste precursor production (Table 2). Plant-derived compounds, like polyphenols and terpenoids, for example, can activate genes involved in producing volatile molecules, which contribute to pleasant meat tastes. Cristo et al. (2022) found that supplementing broiler chicken diets with certain plant extracts enhanced the expression of genes involved in the synthesis of aroma compounds, resulting in improved meat flavor. Scientists, for example, can use nutrigenomics knowledge to develop diets that stimulate the expression of genes involved in vitamin D production in animals, resulting in meat products with higher vitamin D levels (Carlberg, 2019). This novel technique has the potential to change the livestock sector by providing meat products that meet both consumer tastes and nutritional needs.

**Table 2:** Nutrigenomics and meat quality attributes in livestock.

Species/system	Nutritional intervention	Omics layer	Outcome trait	Summary of nutrigenomic effect	Effect size / quantitative change	Range of improvement	Study-level context	References
Beef cattle	Protein/energy supply affecting muscle development pathways	Transcriptomics/nutrigenomics	Tenderness	Modulates genes involved in muscle development, collagen deposition, and proteolysis.	Quantitative improvement in tenderness was not consistently reported in the cited review-level sources.	NR	Mechanistic evidence focused on gene-expression pathways related to tenderness.	(Alagawany et al., 2022a; Hocquette et al., 1998)
Beef cattle	Dietary modulation of muscle fibre characteristics	Transcriptomics / nutrigenomics	Tenderness	Alters muscle fibre structure and composition, influencing final meat tenderness.	No common pooled effect size reported across the cited sources.	NR	Narrative evidence linking diet-responsive muscle biology to meat texture.	(Alagawany et al., 2022a; Hocquette et al., 1998)
Livestock meat systems	Dietary manipulation of intramuscular fat and fatty acids	Lipidomics/nutrigenomics	Flavor	Influences intramuscular fat deposition and fatty-acid profile, thereby altering flavor perception.	Magnitude of change depends on diet composition and species; no single comparable effect size reported.	Variable by species and diet	Comparative evidence across livestock meat-quality studies.	(Hiller et al., 2011; Wood et al., 2008)
Livestock meat systems	Nutritional strategies increasing desirable flavor precursors	Nutrigenomics / meat science	Flavor	Contributes to desirable flavor attributes through altered nutrient partitioning.	Quantitative flavor-score changes were not consistently extractable from the cited sources.	NR	Evidence synthesized qualitatively from meat-quality literature.	(Hiller et al., 2011; Wood et al., 2008)
Livestock meat systems	Dietary modulation of lipid metabolism and aroma-related pathways	Transcriptomics / lipid metabolism	Flavor	Changes expression of genes associated with lipid metabolism and aroma compounds.	No standardized cross-study effect size available in the cited sources.	NR	Mechanistic interpretation of nutrigenomic effects on aroma-related metabolism.	(Hiller et al., 2011; Wood et al., 2008)
Livestock meat systems	Dietary fortification and nutrient-balancing strategies	Nutrigenomics / nutrient profiling	Nutritional content	Affects levels of vitamins, minerals, amino acids, and beneficial fatty acids in meat.	Quantitative values differ markedly according to species and fortification strategy; not pooled in the cited reviews.	Variable by nutrient and feeding strategy	Comparative evidence on nutrient composition and meat quality.	(Castellini et al., 1998; Scollan et al., 2014)
Livestock meat systems	Precision feeding for nutrient-dense meat	Nutrigenomics / precision nutrition	Nutritional content	Improves the nutritional profile of meat for consumers.	Effect sizes not uniformly reported in a way that allows direct synthesis.	NR	Review-level conclusion based on heterogeneous intervention studies.	(Castellini et al., 1998; Scollan et al., 2014)

*Note*: Quantitative data are reported wherever explicitly available from the cited source(s). “NR” indicates that no directly extractable quantitative effect size or improvement range was reported in the cited study/review as used in this manuscript.

For milk production, traditionally, livestock diets were developed based on broad nutritional requirements. These approaches, however, frequently fail to take into account the individual genetic variations that play a critical role in how animals absorb and utilize nutrients. Nutrigenomics may help address this constraint by integrating information on the genetic variability among animals, which could enable more precise dietary strategies as genomic technologies become increasingly accessible (Fenech et al., 2011; Ronteltap et al., 2013; Sales et al., 2014). The use of nutrigenomics in livestock has been proven to improve milk quality (Bauman et al., 2006; German et al., 2011). According to research, personalized diets can enhance milk fat content, fatty acid profile, and protein composition (Gallardo & Teixeira, 2023; Nowacka-Woszuk, 2020). Researchers, for example, have successfully used nutrigenomic techniques to boost the concentration of essential omega-3 fatty acids in milk, which have been linked to a variety of health benefits for consumers (Bionaz et al., 2015; Nowacka-Woszuk, 2020). Furthermore, nutrigenomics has been associated with a reduction in undesirable milk components such as saturated fats (Leroux et al., 2016).In the poultry sector, the application of nutrigenomics has yielded encouraging results in terms of changing egg composition and quality through targeted nutritional interventions that interact with the birds’ genetic makeup (Das et al., 2018). Changing the dietary omega-3 fatty acid content, for example, it has been shown to improve the deposition of these beneficial fatty acids in the yolk, thereby increasing the nutritional value of eggs (Jiang et al., 2023). Similarly, nutrigenomic insights can lead to the enrichment of eggs with substances such as selenium and vitamin E, which contribute to improved egg quality and consumer health (Muhammad et al., 2021). Specific amino acid such as methionine, inclusion in the diet, as suggested by genetic predisposition, can increase eggshell quality and strength (Gao et al., 2024). Furthermore, manipulating vitamin D metabolism using nutrigenomic techniques has shown promise in increasing eggshell thickness, which is an important element in determining egg quality (Gao et al., 2024). Optimizing reproductive performance

Nutrigenomics has emerged as a promising approach for improving reproductive performance in livestock by elucidating how dietary components regulate gene expression and reproductive functions. Nutrigenomics enables us to understand how individual nutrients and dietary components, particularly those associated with reproductive functions, can alter gene expression. Micronutrients such as folate, vitamin D, and zinc, for example, are known to alter gene expression in both male and female animals, influencing gametogenesis, hormonal regulation, and overall fertility (Abdelatty et al., 2018; Bach, 2018). Furthermore, epigenetics, a crucial component of nutrigenomics, plays an important role in controlling gene expression without changing the DNA sequence. Dietary variables can induce epigenetic changes, such as DNA methylation and histone acetylation, which have been associated with reproductive performance in livestock (Sinclair et al., 2007). In poultry, precision nutrition approaches are particularly relevant for mitigating fertility decline associated with aging and environmental stressors. For example, the supply of methyl donors such as methionine and betaine has been shown to regulate the methylation of reproduction-related genes in laying hens, thereby improving egg quality. In males, supplementation with n-3 polyunsaturated fatty acids (PUFAs) and antioxidants, including vitamin E and selenium, enhances sperm quality and motility by protecting germ cells against oxidative stress (Fouad et al., 2020; Hassan et al., 2022; Naeem & Fatima, 2025). Similarly, it can improve egg quality and promote a healthier reproductive tract environment in females, lowering the chance of embryo loss and enhancing overall reproductive success (Leroy et al., 2008).

Proper feeding throughout critical periods can result in beneficial epigenetic alterations that boost fertility (Table 3). Furthermore, nutrigenomics allows for the creation of individualized nutrition regimens for particular animals or breeds depending on their genetic characteristics. We can improve nutrient intake to maintain reproductive health and overcome genetic predispositions to reproductive problems by adapting diets to each animal’s particular genetic makeup (Kizilaslan et al., 2022). Nutrigenomics research in male livestock has shown that nutritional interventions can increase sperm quality, motility, and viability by altering genes involved in spermatogenesis (Qu et al., 2019). Many livestock species are predisposed to reproductive problems, such as polycystic ovarian syndrome (PCOS) in cattle and low sperm counts in pigs. Nutrigenomics, which addresses genetic susceptibilities through targeted dietary treatments, has the potential to reduce the occurrence of such illnesses (Urbański et al., 2021). Improving reproductive performance through nutrigenomics may reduce the resources required to sustain breeding herds, decrease environmental impacts and lower production costs, while enhancing the overall profitability for cattle producers (Osorio & Moisa, 2019).

**Table:** 

Livestock Species	Nutrigenomic Impact	References
Cattle	Improved oocyte quality and embryo development	(O & Boland, 1999)
Swine	Enhanced litter size and reduced pregnancy-related complications	(Costa et al., 2019; Da Silva et al., 2016)
Sheep	Better reproductive efficiency	(Ferguson & Barnett, 2012; Hassan et al., 2021)
Poultry	Increased egg production and fertility rates Enhanced sperm quality and motility by protecting germ cells against oxidative stress	(El-Sabrout et al., 2023; Fouad et al., 2020)
Goats	Improved kidding rates and healthier offspring	(Martyniuk, 2020; Osorio et al., 2017)

**Table 3:** Impact of nutrigenomics on reproductive performance in livestock species.

Species/system	Nutritional intervention	Omics layer	Outcome trait	Summary of nutrigenomic effect	Effect size / quantitative change	Range of improvement	Study-level context	References
Cattle	Energy and nutrient management during the peri‑conception period	Reproductive nutrigenomics / physiology	Oocyte quality and embryo development	Improves follicular environment, oocyte competence, and embryo survival when nutrition is appropriately managed.	The cited review reports directionally important effects, but not a single pooled effect size suitable for tabulation.	Variable by nutritional plane and reproductive stage	Review of nutritional effects on ovulation, embryo development, and pregnancy establishment in ruminants.	O'Callaghan & Boland, 1999
Swine	Maternal nutrient supply and reproductive-support diets	Nutrigenomics / reproductive physiology	Litter size and pregnancy outcomes	Associated with improved litter performance and reduced pregnancy-related complications.	Quantitative responses differ among parity classes and supplementation protocols in the cited studies.	Variable by study design	Evidence synthesized across reproductive and maternal nutrition studies in swine.	Costa et al., 2019; Da Silva et al., 2016
Sheep	Targeted feeding during breeding and early gestation	Nutrigenomics / reproductive management	Reproductive efficiency	Supports improved conception and reproductive efficiency through diet-sensitive physiological pathways.	No single comparable effect size was reported across the cited sources.	NR	Species-specific evidence remains heterogeneous and mostly context-dependent.	Ferguson & Barnett, 2012; Hassan et al., 2021
Poultry	Antioxidant and reproduction-support supplementation	Nutrigenomics / oxidative-stress biology	Egg production, fertility, and sperm quality	Supports higher egg output and fertility while protecting germ cells against oxidative stress.	Quantitative gains vary by additive, dosage, and flock conditions; the cited review evidence is heterogeneous.	Variable by additive and management conditions	Evidence integrates reproductive and oxidative-stress studies in poultry.	El-Sabrout et al., 2023; Fouad et al., 2020
Goats	Maternal nutrition and transcriptome-informed reproductive support	Transcriptomics / reproductive nutrigenomics	Kidding rate and offspring health	Associated with improved kidding success and healthier offspring.	Quantitative effect sizes not consistently extractable from the cited sources.	NR	Mechanistic and review evidence on nutrigenomic regulation in small ruminants.	Martyniuk, 2020; Osorio et al., 2017

*Note*: Quantitative data are reported wherever explicitly available from the cited source(s). “NR” indicates that no directly extractable quantitative effect size or improvement range was reported in the cited study/review as used in this manuscript.

## 8 Genetic selection and breeding programs

**Fig. 3:**
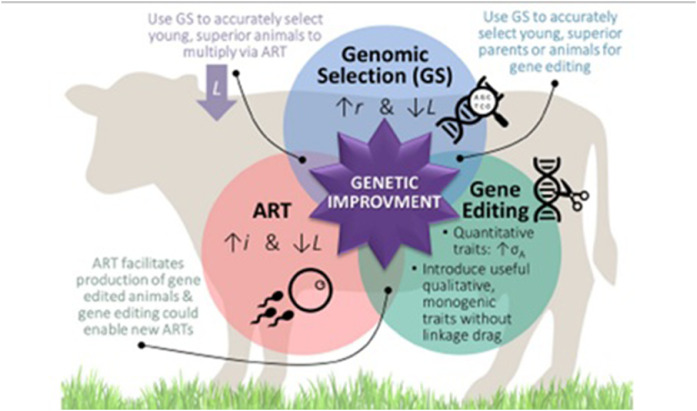
Synergistic relationships between genomic selection (GS), assisted reproductive technologies (ART), and gene editing for the genetic improvement of cattle (Mueller & Van Eenennaam, 2022).

When applied to livestock, nutrigenomics has the potential to significantly improve genetic selection and animal output (Kader Esen et al., 2022). This method enables breeders to make more educated judgments regarding which animals to breed, resulting in healthier, more productive cattle (Kader Esen et al., 2022; Loor, 2022). Nutrigenomics enables scientists to identify individual genes involved in how animals process nutrition. Fan et al. (2010), for example, used nutrigenomics to find genes in pigs related to increased feed efficiency, which is critical for increasing livestock production. According to Mueller and Van Eenennaam (2022), synergistic relationships between Genomic Selection (GS), Assisted Reproductive Technologies (ART), and Gene Editing hold enormous promise for the genetic improvement of cattle, allowing for accelerated progress in breeding programs (Fig. 3). They showed that the synergy between these three components is evident in their collective ability to maximize genetic gains. Breeders can improve an animal’s development and health by understanding its genetic tendency for nutrition use. Ariyarathne et al. (2021) show how nutrient utilization-based genetic selection can help reduce nitrogen waste in beef cattle and then reduce their environmental impact. Nutrigenomics can contribute to the production of disease-resistant animals. Nutrigenomics-guided genetic selection can result in livestock with better meat quality attributes. Durunna et al. (2011) investigated how nutrigenomics-informed genetic selection can save livestock producer’s money by lowering feed costs and increasing production efficiency. Nutrigenomics is a significant method for genetic selection improvement in cattle because it identifies genes related to nutrient utilization, customizes diets, reduces environmental impact, improves disease resistance, improves meat quality, and delivers economic benefits.

## 9 Economic benefits

Nutrigenomics enables livestock sectors to improve their production, health, and economic viability. Nutrigenomics aids in the identification of specific dietary components and formulations that maximize feed efficiency. Producers can reduce feed waste and overall production costs by customizing diets to an animal’s genetic composition. For example, nutrigenomics-guided diets enhanced feed conversion rates in broiler hens, resulting in significant cost savings (Gao et al., 2025). Preliminary evidence suggests that nutrigenomic approaches may contribute to improved disease resilience by identifying diet–gene interactions involved in immune regulation. Cao et al. (2024) found that gene-based nutritional therapies enhanced the animals’ immune defenses, lowering the prevalence of common illnesses in dairy cattle. Nutrigenomics has the potential to find diet-related elements that improve reproductive performance, leading to larger and healthier litters or offspring. Tohumcu and Tulan Tohumcu (2024) found that personalized feeding based on genetic profiles enhanced fertility rates and lowered the cost per successful pregnancy in dairy cows. Nutrigenomics can help improve the sustainability of cattle production techniques. It is feasible to reduce the environmental footprint of livestock farming by optimizing diets, which reduce greenhouse gas emissions and the use of natural resources such as water and land (Jiang et al., 2020). Thus, nutrigenomics integration in animal production offers great economic potential. Increased feed efficiency, improved growth rates, disease resistance, better reproductive performance, and sustainable resource management are some of the primary benefits that can translate into significant cost savings and increased profitability for the cattle sector (Gebremikael et al., 2018; Jiang et al., 2023).

Conclusion Nutrigenomics is changing the way farmers approach feed formulation by linking genetics, nutrition, and animal production, thereby transforming livestock and poultry management strategies. By incorporating genetic information into the process, producers can develop personalized diets adapted to the genetic characteristics of animals, leading to improved growth performance, health, and environmental sustainability. As nutrigenomics knowledge grows, this approach has the potential to improve animal production efficiency while contributing to resource efficiency, waste reduction, and global food security.

Furthermore, nutrigenomics allows a deeper understanding of individual dietary responses, facilitating the identification of genetic traits associated with disease resistance, growth efficiency, and nutrient consumption. This understanding paves the way for selective breeding techniques that result in healthier, more resilient, and economically sustainable animal herds. Additionally, nutrigenomics interventions may help mitigate the effects of certain genetic predispositions that increase susceptibility to nutrient-related disorders. Although further research is required to facilitate the large-scale implementation under commercial conditions, nutrigenomics represents a promising pathway toward more resilient, efficient livestock and poultry production systems.

## 10 Addressing nutritional challenges

Understanding how animals’ genetic makeup interacts with their diet has the potential to transform the livestock sector by improving animal health, productivity, and the nutritional quality of animal products (Haq et al., 2022). However, in order to fully fulfill its potential, this intriguing industry must overcome a number of nutritional difficulties. The genetic heterogeneity among individual animals within a population is one of the key problems in nutrigenomics research in livestock. Because of genetic differences, different animals may react differently to the same nutritional components (Alagawany et al., 2022a; Bishop & Woolliams, 2014). When establishing nutrition programs for large-scale livestock production systems, these variances can be difficult to account for (Adabi et al., 2011). Nutrigenomics has discovered a complex and multidimensional relationship between genes and nutrition. It is a difficult effort to identify specific genes that regulate food metabolism and response (Adabi et al., 2011). The expression of these genes can be altered by various factors, like nutrition, age, and environmental conditions, making precise dietary recommendations challenging (Kaput et al., 2007). The implementation of nutrigenomic and gene-based nutritional strategies in livestock production also raises ethical, regulatory, and societal considerations. Consumer acceptance of genomic interventions in animal production remains variable and is influenced by perceptions of food safety, naturalness, and trust in biotechnology-based food systems (Rodriguez-Villamil et al., 2024). Moreover, regulatory frameworks governing gene-edited animals differ significantly across regions, while concerns related to animal welfare and equitable access to advanced genomic technologies may affect the adoption of nutrigenomic innovations. Therefore, responsible implementation of these approaches requires transparent governance and careful consideration of societal expectations (Garas et al., 2014; Rodriguez-Villamil et al., 2024).

Another problem is integrating massive volumes of data from genomes, transcriptomics, proteomics, and metabolomics. It can be difficult and time-consuming to analyze and interpret this data to provide effective dietary recommendations for livestock farmers (Goddard, 2009). Implementing nutrigenomics-based livestock production strategies can be costly, especially for small-scale producers. The high expense of genetic testing and customized diets can limit access to new ideas, thereby worsening industrial gaps (Qanbari et al., 2014). Nutrigenomics provides enormous promise for improving animal nutrition and output. On the other hand, addressing the nutritional issues connected with this field is critical for its successful incorporation into the livestock industry.

## 11 Future directions

Looking ahead, the fusion of AI with multi-omics data promises real-time, genetics-customized feeds that could transform farm management, think predicting a 16% drop in herd methane emissions or a 28% jump in vitamin absorption, as shown in early trials (de Oliveiraet al., 2024; Qiu et al., 2022). Pilot systems are already modeling how microbiomes, diets, and genes interact, with applications scaling from lab-grown meat simulations to live poultry flocks in 2025 studies. On the microbiome and epigenetics front, synthetic biology is set to craft next-generation probiotics that fine-tune gene activity, building resilience against climate extremes like heat or drought (Liu et al., 2026). Yet hurdles remain, from ethical debates over gene editing to proving these work at commercial scale (Khan et al., 2024). By 2030, nutrigenomics could cut antibiotic use by half through diets that supercharge natural immunity and trim greenhouse gases 20% via smarter rumen engineering (Zhou et al., 2022).

## 12 Conclusion

Nutrigenomics stands at the exciting intersection of genes, diet, and farm realities—transforming how we feed livestock and poultry from guesswork to precision. From early microarray glimpses of diet’s genomic ripples to today’s multi-omics atlases boosting dairy yields 15% and CRISPR edits slashing methane 16–20%, this field delivers tangible wins: 56% less poultry waste, 45% GHG cuts with smart additive pairings, and 20% efficiency gains without sacrificing output (De Verdal et al., 2013; Dijkstra et al., 2025; Wang et al., 2025). Challenges remain, scaling costs, ethical editing, regulatory paths—but as AI predictions sharpen and sequencing plummets, nutrigenomics isn’t a magic fix; it’s livestock’s next evolution, turning animals into efficient climate partners while feeding our world sustainablylivestock’s next evolution, turning animals into efficient climate partners while feeding our world sustainability.

## Funding

This review received no external funding.

## Institutional review board statement

Not applicable.

## Informed consent statement

Not applicable.

## Ethics statement

This article does not contain any studies involving human participants or animals performed by any of the authors.

## CRediT authorship contribution statement

**Ikram Ben Souf:** Writing – original draft, Validation, Conceptualization. **Mariem Saidani:** Writing – review & editing. **Lotfi Mhamdi:** Writing – review & editing. **Cyrine Darej:** Writing – review & editing, Supervision. **Manel Ben Larbi:** Writing – review & editing. **Naceur M’Hamdi:** Writing – review & editing, Validation, Supervision.

## Declaration of competing interest

The authors declare that they have no known competing financial interests or personal relationships that could have appeared to influence the work reported in this paper.
